# Transactions of the So. Surg. & Gynecological Association

**Published:** 1889-09

**Authors:** 


TRANSACTIONS OF THE SOUTHERN SURGICAL AND GYNECOLOGICAL ASSOCIATION.—Vol. I. Session of 1888, Birmingham, Dec. 4th to sixth. Organized 1887. Birmingham, Ala., Com. of Publication, W. E. B. Davis, M. D., Birmingham, Ala.; W. B. Rogers, M. D., Atlanta, Ga.

Full discussions are not given in the present book, but hereafter it is proposed to have a stenographer to make full reports.

Although the first meeting of the new Association, nearly one hundred members are reported, of these Georgia had 13 representatives; Ala. 27; Tenn. 12; Miss. 5; La. 3, Texas 8; Va. 8; N. C. 4; S. C 5; Ky. 5; Ark. 2

The officers elect for the years '88 and '89 are as follows: President, Hunter MdGuire, M. D., L. L. D., Richmond, Va. Vice Presidents, W. O. Roberts, M. D., Louisville, Ky.; Bedford Brown, M. D., Alexandria, Va. Sec'ty, W. E. B. Davis, M. D., Birmingham, Ala. Treasurer, H. P. Cochran, M. D., Birmingham, Ala. Judicial Counsil, John S. Cam, M. D., W. T. Briggs, M. D., J. M. Taylor, M. D., DeSausure Ford, M. D., V. O. Hardon, M. D.

A cursory glance at the contents of the volume impressed us favorably with the ability and interest of a number of papers. We regret that our space will not permit us to particularize certain of the papers which deserve special mention.

